# Providing information about options in patient decision aids

**DOI:** 10.1186/1472-6947-13-S2-S4

**Published:** 2013-11-29

**Authors:** Deb Feldman-Stewart, Mary Ann O’Brien, Marla L Clayman, B Joyce Davison, Masahito Jimbo, Michel Labrecque, Richard W Martin, Heather Shepherd

**Affiliations:** 1 Division of Cancer Care and Epidemiology, Queen’s University Cancer Research Institute, Level 2, 10 Stuart St. Kingston, Ontario K7L 3N6, Canada; 2 Knowledge Translation Research Network (KT-Net), Health Services Research Program, Cancer Care Ontario and Ontario Institute for Cancer Research and Department of Family and Community Medicine, 500 University Avenue, Fifth Floor, Toronto, Ontario M5G 1V7, Canada; 3 Division of General Internal Medicine and the Robert H. Lurie Comprehensive Cancer Center, Northwestern University, 750 North Lake Shore Drive, 10th Floor, Chicago, Illinois 60611, USA; 4 College of Nursing, University of Saskatchewan, 107 Wiggins Road, Saskatoon, Saskatchewan S7N 5E5, Canada; 5 Department of Family Medicine, University of Michigan, 1500 E Medical Center Drive, Spc 5474, Ann Arbor, MI 48104, USA; 6 Research Center of the Centre hospitalier universitaire de Québec, Saint-François d'Assise Hospital, 10 rue de l'Espinay, D6-728, Quebec City (QC), G1L 3L5, Canada; 7 College of Human Medicine, Michigan State University, 1155 East Paris Ave SE Suite 100, Grand Rapids, MI 49546, USA; 8 Sydney School of Public Health, The University of Sydney, Sydney NSW 2006, Australia

## Abstract

**Background:**

Legal, ethical, and psychological arguments indicate that patients need to receive information about their health situations before their care decisions are made. Patient decision aids (PtDAs) are designed to help patients make decisions; therefore, they should provide information that results in patients understanding their health situation. We reviewed studies that assessed the impact of PtDAs on patient knowledge and on their feeling of being uninformed.

**Methods:**

Our data sources were a published Cochrane Collaboration review that included randomized controlled trials (RCTs) published before 2010 and a systematic review we conducted of RCTs published in 2010. We included trials that compared 1) PtDAs to usual care, and 2) PtDAs with simple information to PtDAs with more detailed information. Outcomes included patients' knowledge and their feeling of being uninformed. Data were analyzed quantitatively and qualitatively. Meta-analyses of similar studies estimated the size of differences.

**Results:**

Thirty-nine RCTs compared a PtDA to usual care and all showed higher knowledge scores for patients in the PtDA groups; a meta-analysis estimated the advantage at 14 (of 100) points. Sixteen (of 39) studies used the Feeling Uninformed subscale; a meta-analysis estimated a reduction of 7 (of 100) points in the PtDA group over usual care. Twenty-one studies compared simple- to more-detailed information in PtDAs. There was a small overall advantage for more detailed information on knowledge scores**;** a meta-analysis estimated the advantage at 5 (of 100) points. Only one study found higher mean knowledge scores for simpler information. Nine (of 21) studies reported using the Feeling Uninformed subscale and a meta-analysis suggested a reduction of 3 (of 100) points for the more-detailed PtDAs over those with simpler information. Only one study found that simpler information resulted in patients feeling more informed.

**Conclusions:**

It appears that PtDAs result in patients having higher knowledge scores and in reduced feelings of being uninformed over patients who receive usual care. It also appears that PtDAs with more detailed information generally result in slightly higher knowledge and lower “Feeling Uninformed” scores than those with simpler information, but the differences are small and can be reversed under some circumstances.

## Background

For patients to make decisions about their care, they need to have knowledge about their health situations and the options being offered to them. For patients to have this knowledge, they must be provided with relevant information. Since patient decision aids (PtDAs) are intended to help patients make decisions about their care, a core aspect of their design is the provision of that relevant information.

### A definition

The selection of the relevant information that should be included in PtDAs is guided by informed consent requirements as well as by patient reports about the information that they need. Thus, the relevant information in PtDAs should include:

• How the untreated condition is expected to develop.

• The procedures involved in each treatment option.

• The potential benefits -- including their likelihoods -- of each treatment option.

• The side effects and potential harms -- including their severity and their associated likelihoods -- of each treatment option.

• For screening or diagnostic tests …

○ the frequency of true/false positive and true/false negative results, and

○ the recommended follow-up actions that could include treatment options for true positive results

• In addition, patients’ information needs that are outside these content areas also need to be identified and addressed.

### Purpose

The purpose of this paper is to present the theoretical justification for evaluating PtDAs according to the quality dimension of *‘providing information about options’*, to review the current empirical evidence for such evaluation, and to identify and discuss some emerging theoretical, evidentiary, and research issues about the provision of information in PtDAs.

## Theoretical justification for evaluating patient decision aids on this quality dimension

The argument that patients need to have knowledge in order to make a decision—and, hence, the argument that information should be provided in a PtDA—has several theoretical foundations. Below, we touch on two such foundations: the ethical and legal imperatives of informed consent, and the ways in which decision-making theories highlight patient information needs.

### Ethical and legal obligations of informed consent

Healthcare providers are bound by the *ethical* doctrine of informed consent, which is founded on three principles: (1) autonomy (which obligates the providers to ensure that the patients can act in their own best interest without undue pressure); (2) beneficence and non-malfeasance (which obligates the providers to choose to do good and to avoid doing harm to patients); and (3) distributive justice (which obligates the providers to treat all patients equally) [1,2]. The implication of these ethical principles is that healthcare providers need to ensure that all patients have equal access to the information deemed important to the decision.

In most jurisdictions, there is a *legal* obligation of informed consent. This obligation makes healthcare providers responsible for ensuring that patients understand their condition, all available tests or treatments--including doing nothing--and each treatment’s potential benefits and harms.

In the context of informed consent, specifying which details to cover has built on legal precedent, making use of “standards”. Three standards have emerged over time, each attempting to compensate for deficiencies of the previous one. The first was the “Professional” Standard, which refers to the information that doctors agree should be provided. When the Professional Standard did not capture what was important to patients [3], it was replaced with the “Objective” Standard, that which a "reasonable" person in the patient's position would need to be provided (as decided by a jury). Differences between patients in what they consider important to the decision has led to the third standard, the “Subjective” Standard, that which the individual patient deems important to the decision [1]. Intrinsic to the standards is that the information should be evidence-based as much as possible, using the best quality evidence available [4] (see also in this special supplement: Montori et al., "Basing information on comprehensive, critically appraised, and up-to-date syntheses of the scientific evidence").

### Decision-making theories highlight patient information needs

Prescriptive theories of decision making (e.g., Expected Utility Theory [5]) and descriptive theories of decision making (e.g., Behavioral Decision Framework [6]; Conflict Model [7]; Differentiation and Consolidation [8]; Fuzzy Trace Theory [9]; Image Theory [10]; Parallel Constraint Satisfaction [11]; and Search for Dominance Structure [12]) all suggest that, to make a decision, the decision maker needs a) to develop a knowledge base that’s relevant to the decision problem, and then b) to establish their knowledge-based preferences for particular aspect(s) of the options or for an option as a whole. Therefore, a wide range of psychological theories imply that patients contemplating a health care decision need to be provided with information from which to derive a relevant knowledge base and, thereafter, upon which to formulate their preferences. However, while some information may be clearly relevant to all decision makers for a given decision, the relevance of some other aspects of information may vary from person to person.

The implication of all these theories is that, for them to make decisions, patients need information that is relevant to their individual needs. This implication creates a particular challenge for PtDA designers because empirical evidence suggests that patients’ information needs for decision making often differ from the information priorities of their healthcare providers [13], and furthermore, that the information needs often vary considerably from one patient to the next [14-16].

## Empirical evidence for evaluating patient decision aids on this quality dimension

The theoretical underpinnings outlined above indicate that patients facing health care decisions need to be provided with appropriate information so that they have the knowledge required to arrive at preference-based choices. One could argue that, in regular clinical practice alone, patients already receive such information and already use it to formulate the knowledge base they need to make choices. If this is so, then the use of PtDAs to help in the process of information provision is, in effect, moot, and, by extension, it’s not worth including the quality dimension of ‘*providing information about options*’ in the roster of evaluative dimensions used to assess the adequacy of a PtDA. Therefore, we asked the first-order review question: “What is the quality of the evidence that PtDAs augment patients’ knowledge?”

(Note that it is beyond the scope of this paper to present overviews of additional second-order review questions. Such second-order reviews could include, for example, examinations of a) the empirical evidence about whether or not PtDA developers conduct appropriate preliminary studies to identify patient information needs that exceed the requirements of Informed Consent, or b) the empirical evidence about the extent to which current PtDAs actually provide the content that meets Informed Consent requirements (e.g., [17]. These would, indeed, be important additional review topics to undertake, if, in fact, the empirical evidence indicates that PtDAs, in general, foster higher levels of patient knowledge.)

### Sources and strategies

In the sections below, we synthesize the results of two systematic reviews. One is a Cochrane Collaboration review of the published reports about the effects of PtDAs tested in randomized controlled trials (RCTs) up to the end of 2009 [18] This Cochrane Collaboration’s review identified 50 such RCTs.

The other review supplemented the first; we identified 2010 publications reporting PtDA RCTs up to the end of 2010 by searching OvidSP, including all databases (e.g., Medline, AMED, EBM Reviews, EMBASE, Global Health, Ovid Healthstar, PsycExtra, PsychInfo), and using the search terms: (“decision aid” or “decision support” or “decision making”) AND (“randomized trial” or “controlled trial” or “comparison”). This 2010 search identified an additional 10 trials. All 60 of these reports of RCTs evaluating the effect of PtDAs on knowledge involved patients facing actual choices.

In examining data, we focused on two types of studies : 1) interventions in which a patient uses a PtDA compared to patients who receive usual care, and 2) PtDAs with simple- as compared to more-detailed information. Within each type of study, we focused on two outcomes related to the effects of providing information about options: objective patient knowledge scores; and subjective self-reports of how well-informed patients feel.

### PtDAs versus usual care

Of the 60 RCTs, 39 compared a PtDA to “usual care”, which we defined as no intervention beyond that usually given in the study setting.

#### Effects on objective knowledge scores

Among these 39, the 2011 Cochrane Collaboration review [18] included a meta-analysis of 26 studies that compared PtDAs to usual care in terms of the effects on knowledge scores. It found that patients using PtDAs had mean knowledge scores that were, on average, 14 out of 100 points higher (95% CI: 11 to 16) than the mean scores of patients who received usual care. This Cochrane Collaboration review also identified an additional 8 studies that could not be included in the meta-analysis. Of these, 5 reported statistically significant improvement in knowledge scores in PtDA groups compared to usual care, although one [19] showed the advantage only if the PtDA was used during the consultation (and not when it was used prior to the consultation). The other 3 studies reported a statistically significant improvement from baseline for DA groups.

Our supplementary review identified five additional trials published in 2010 that compared PtDA to usual care in terms of the effects on knowledge scores. All 5 reported statistically significantly improved knowledge for the PtDA group. Two reported higher mean knowledge scores [20,21]; 2 reported larger mean improvement from baseline scores [22,23]; and the fifth study reported that a larger percentage of PtDA patients improved their knowledge scores and that there was a statistically significant larger mean improvement in scores for the PtDA group [24].

Thus, overall, it appears that providing patients with a PtDA results in higher objectively-assessed knowledge scores than those who receive usual care.

#### Effects on subjective “feeling informed” scores

Most frequently, patients’ subjective reports have been measured by the “Feeling Uninformed” subscale of the Decisional Conflict Scale (DCS, [25]). The 2011 Cochrane Collaboration review [18] identified 16 studies that reported observations obtained on the Decisional Conflict Scale “Feeling Uninformed” subscale, and a meta-analysis of those data found a 7-point (out of 100 points) reduction in reports of feeling uninformed (95% CI: -9 to -4). (In our supplementary review of the five additional studies identified in 2010 that compared PtDAs versus usual care, none reported their observations on the “Feeling Uninformed subscale”, even when the investigators had used the Decisional Conflict Scale.)

Evidence gathered using objectively-assessed knowledge scores is currently considered the “gold standard” measure of information comprehension. However, the subjectively-gathered data reported in the 2011 Cochrane Collaboration review imply that providing patients with a PtDA also results in those patients feeling as if they are better informed than those who received usual care.

### Simple versus more detailed PtDAs

Twenty-one of the 60 trials compared a simpler to a more-detailed PtDA; we defined “more-detailed” as providing information (with or without other components added to the PtDAs) that was not provided in the “simple” PtDA.

#### Effects on objective knowledge scores

The 2011 Cochrane Collaboration review [18] included a meta-analysis of 14 trials that compared simple to more-detailed PtDAs and found that, on average, the more-detailed decision aids resulted in a 5-point (out of 100 points) improvement in mean knowledge scores beyond the gains in knowledge scores induced by the simple PtDA (95% CI: 3 to 7), which is considered a small effect. One study that could not be included in this analysis found no difference between the groups [26].

Our supplementary review identified five studies that compared simple to more-detailed PtDAs. Of these, three found some statistically significant evidence of higher knowledge scores for the more-detailed PtDA. However, among these three, the differences often were limited. One found a higher mean knowledge score [27], one found a significant improvement in mean knowledge scores from baseline in the more-detailed PtDA group but not in the simpler-PtDA group [28], and one found no difference in mean overall knowledge scores, although the more-detailed group had more accurate perceptions of the risks deemed most important to the decision [29]. The fourth study reported no difference in mean knowledge scores between the groups [30]. The final, fifth, study was designed for low-literacy patients, so the intervention PtDA contained less information and used simpler language and graphic illustrations compared to the standard information provided to the control group. The authors reported that the group who received less information (in simpler language with graphics) had higher mean knowledge scores, and that a statistically significantly larger proportion of participants reached the knowledge threshold that the authors defined for “informed decision making” [31].

Thus, it generally appears that the more-detailed PtDAs seem to result in slightly higher knowledge scores than the simpler PtDAs, but the differences are very small, often isolated, and it appears that they can be reversed under some circumstances.

#### Effects on subjective “feeling informed” scores

The 2011 Cochrane Collaboration review [18] included a meta-analysis of the results of 9 studies that compared simple to more-detailed patient PtDAs in terms of patients’ “Feeling Uninformed” scores. The analysis revealed that the more-detailed PtDAs resulted in a slight 3-point (out of 100 points) reduction in feeling uninformed (95% CI: -5 to 0).

Of the five studies comparing simple to more-detailed PtDAs that were identified in our supplementary review, two found that the more-detailed PtDA reduced “Feeling Uninformed” scores significantly more than the simple PtDA [28,29]. In the study of patients with low literacy, the patients who received the PtDA that presented less information in simpler language and that used graphic illustrations reported higher scores on the low-literacy version of the “Feeling Uninformed” subscale (65% versus 52%) [31]. Two of the five studies did not find a difference between the groups’ scores on the DCS “Feeling Uninformed” subscale [27,30]. However, one of those studies did find that a significantly higher percentage of the more-detailed PtDA group reported having enough information to make a decision (89% versus 80%) [27]. Thus, parallel to the knowledge-score comparisons, it appears that more detailed information seems to result in slightly higher “Feeling Informed” scores but the differences are small, often isolated, and it appears they can be reversed under some circumstances.

Taken together, it appears that patients using PtDAs show improved objective knowledge scores and improved feelings about being informed over patients who receive usual care. (It also appears that, typically, more detailed information offers patients a slight benefit over those who receive simple information in their PtDAs, but the advantage is small, not always realized, and can be reversed.) Therefore, the empirical evidence indicates that the use of PtDAs to help in the process of information provision is not a trivial action, and, by extension, it is important to include the quality dimension of ‘*providing information about options*’ in the roster of evaluative dimensions used to assess the adequacy of a PtDA.

## Discussion

This review began by suggesting that the legal and ethical obligations for informed consent, along with prescriptive and descriptive theories of decision making, all argue for providing information about options to patients so that patients have the knowledge they need in order to make care decisions. Therefore, whether or not it’s worthwhile to assess the quality of PtDAs in terms of its information-provision depends on whether or not such PtDAs are actually capable of improving patients’ knowledge. Empirical evidence suggests that this is so – because patients feel more informed and are actually more informed when the information is presented in a PtDA than as part of usual care. The results of comparisons of more-detailed and simpler PtDAs are less clear, but it appears that the more-detailed PtDAs tend to be slightly more helpful to most patients than the simpler ones.

As we have indicated above, for patients to have the information that they require to make a decision, the content provided in a PtDA typically needs to go beyond that which fulfills Informed Consent obligations. Below, we identify some issues that complicate the identification of such content, we present relevant evidence, and we discuss the implications of each information-identification issue. In addition to these complications, what patients actually understand about their decision is affected not only by what information is provided but also by *how* it is provided. Below, we identify some presentation issues, provide evidence relevant to those issues, and highlight where needed evidence is not yet available.

### Content-related issues

As noted above, identifying patients’ information needs is an important aspect of developing PtDAs (see also in this special supplement: Coulter A et al, "A Systematic Development Process for Patient Decision Aids"). Three issues are emerging as important when identifying those needs.

The first issue is that investigations need to drill down to the details that are important, rather than relying on identifying broad categories that patients are interested in, and then assuming that it is clear which details are important. For example, patients with early-stage prostate cancer concerned about “the cancer spreading” can be concerned about the *chances* of the cancer spreading, which is assumed and true for some patients, but some patients are concerned about *where* the cancer will spread (e.g., wanting to avoid brain metastases more than metastases to other parts of the body) [32]. Thus, while it is increasingly accepted that patients’ information priorities often differ from those of their healthcare providers [13,33], the extent of the differences is not yet clear. For each decision, investigation of the detailed information that affects patients’ decisions will help clarify what information should be provided in a PtDA for that decision.

The second issue that is emerging is the wide variability within patient populations in what information they want [15,34,35]. Although a review of PtDAs suggests that developers frequently rely on focus group and/or interviews with patients to identify their needs [17], it is only through systematic quantitative study that variability can be identified. The qualitative studies help ensure depth of understanding, but quantitative study for each decision will help determine (a) which needs are most prevalent, and (b) the extent of variability in particular needs across patients.

The third issue is that information that patients need for decision making can extend beyond that which is necessary to select one option from among those offered. Additional needs include bolstering behavioral intentions (e.g., the Theory of Planned Behavior [36]), bolstering self-efficacy (e.g., the Theory of Reasoned Action [37]), decreasing anxiety or enhancing comfort levels with the decision process or the decision itself, and adjusting processes to match the individual’s style of information-seeking in decision making (e.g., information “blunting” versus “monitoring” [38]). It is important for empirical studies to identify what is required for the broader range of decision-making needs so that they can be addressed. After information for the broader range of needs is presented, the PtDA can explicitly help the patient identify what is important to him/her specifically in selecting their preferred option—a strategy that has been used successfully[39]. Variability in information needs within populations presents particular challenges to PtDA developers. Because PtDAs are intended to help the individual patient with her/his decision, it is important to be able to accommodate wide variability in information needs within a patient population. While there is some evidence for strategies that appear to be effective [40]; [39], further research is needed to determine the best ways of tailoring patient PtDAs to address the information needs of the individual patient, both within and outside busy clinical practices.

### Presentation issues

As suggested above, rigor in identifying what information to provide does not ensure that patients will be able to understand it, and evidence suggests that how the information is presented can affect patients’ comprehension. For example, aspects of text presentation that can affect comprehension include its structure [41,42], layout [43,44], language [45] and font [41] (see also in this special supplement: Abhyankar et al.’s “Balancing the Presentation of Information and Options in Patient Decision Aids: An Updated Review"; and McCaffery et al.’s “Addressing Health Literacy”). While research on many factors affecting comprehension of text is ongoing, much more is needed in the particular context of PtDAs. Presentation of quantitative information—such as the chances of potential benefits and risks—can be affected by aspects including the particular concept being presented (e.g., probabilities versus frequencies) [46], the type of visual format used, and the complexity of information conveyed [47] (see also in this special supplement: Trevena et al.’s “Presenting Quantitative Information about Decision Outcomes: A Risk Communication Primer for Decision Aid Developers”). There is active research around the presentation of quantities in PtDA contexts, and the evidence suggests that many particulars about a given situation will affect which presentations are most easily and accurately understood; for example, when comparing outcomes of two options, the format most effective for judging which quantity is larger (or smaller) differs from the one that is most effective for determining the exact difference between the two [47]. Furthermore, text and numeric presentations can interact to affect comprehension [48]. Moreover, if graphics, including both quantitative and illustrative, do not directly reinforce the textual information, they can distract from core information and reduce recall accuracy [49]. Finally, although evidence presented above suggests that more detailed DAs can generally result in small improvements in the amount of relevant information recalled by patients, there are times when less information results in greater comprehension [50]. See “Population-specific effects”, below, for further information.

In addition to affecting comprehension, presentation format can affect decision-making processes. Presenting text in table format helps readers make direct comparisons, which, in turn, helps decision making [51,52]. It should also be noted that when options are presented sequentially (i.e., one after the other), the order in which they are presented can shift preferences [53]. The order in which pieces of information are presented affects how important patients judge the particular pieces of information to be [33]. Thus, the order of information provided can affect many types of decision processes. Further research is needed to clarify which aspects of presentations affect decision processes and how to address that impact.

While some generalities exist around how information presentation affects its potential to be understood, there is evidence suggesting that some of these effects could be population specific. In addition to literacy levels being important, age may also be an important consideration, such as when using illustrations [54]. For instance, it is possible that older adults may have difficulty integrating illustrations with textual information [45]. Further research is needed to clarify what population-related factors are important considerations for how information is presented. Beyond improving our understanding of population-specific factors that affect comprehension and decision processes, further research is needed to clarify whether particular aspects of clinical situations can affect comprehension and decision processes; for example, are there systematic differences between curative vs palliative decisions that interact with how information is processed, or between reversible and non-reversible decisions?

Presentation issues can go beyond those related to presenting particular pieces of information. Medium-specific considerations can also affect how well information is understood by patients (see also in this special supplement: Hoffman et al.’s “Delivering Patient Decision Aids on the Internet”). For example, multi-media presentation can result in poorer comprehension than when the information is presented in a single medium, such as presenting auditory and text messages that are identical compared to the text by itself. Using different media to complement each other's messages, however, can improve comprehension [55], such as presenting a drawing that reinforces the main message of text. Evidence from a systematic research program on multi-media learning provides guiding principles on how to maximize the effectiveness of multi-media presentations [56]. Multimedia can be used to implement “entertainment education,” which has resulted in higher knowledge gains for low literacy patients than audio-booklet presentation; however, it has no apparent differential impact on high-literacy patients [26].

We note that, while all of the above (and more) aspects of presentation can affect what patients understand, it is not clear how they affected the RCTs that led us to conclude that PtDAs over usual care, and more-detailed over simpler PtDAs, result in improved patient knowledge. The one potential exception is the low-literacy PtDA, where its results appear to reverse the trend seen in most other simpler versus more-detailed comparisons. The study population was also different, however, which complicates interpretation of the results.

### Additional issues

Issues related to information in PtDAs extend beyond defining and presenting the information. As noted above, recall, which demands that patients store information in memory that can be retrieved at a later time, has been typically used to assess effectiveness of information presentations. We suggest that further development is required around which outcomes should be used to assess effectiveness of presentations and when they should be assessed. If the goal of PtDA is to assist decision making, does a patient’s inability to recall information that does not affect their particular decision mean that the PtDA has not help them arrive at a well informed decision? We suggest that understanding the information that is provided is a necessary prerequisite for the PtDA to be helpful. But, recall goes beyond comprehension, and evidence is clear that it is affected by its own host of factors [57]. We, therefore, suggest that in addition to PtDA evaluations being guided by decision-making theory [58], there should be meta-theoretical considerations (i.e., theoretical thinking *beyond* the decision making theory) around which outcomes to assess and when (see also in this special supplement: Stacey et al.’s “Coaching and Guidance with Patient Decision Aids: A Review of Theoretical and Empirical Evidence”).

Observations and conclusions presented here are limited in a number of ways. We relied on the Cochrane Collaboration review [18] for assessments of the quality of the studies they included in their review. In addition, we restricted all studies to RCTs to rely on the highest level of evidence but do recognize that there are numerous non-randomized studies that can provide some insights into relevant issues. Further, all the studies we relied on were conducted in English, in Western countries. Further research is needed to clarify language and cultural issues within those countries, and what can be generalized beyond them. Finally, issues relevant to information presentation that we have identified in this paper come from very broad educational and psychological literatures, where further guidance can be found.

## Conclusions

Information about options is an important dimension of PtDAs and its provision in PtDAs typically results in improved knowledge in patients over usual care. More detailed information can improve that knowledge slightly more than simple information. How the information is presented, however, can have a large impact on the knowledge patients acquire, by affecting patient ability to understand and integrate the information.

Defining the content to include in a particular PtDA requires consultation with relevant healthcare providers and the targeted patient population, using both qualitative and quantitative approaches. The combination of the two types of studies will ensure that developers have both breadth and depth of understanding of the information required and it will reveal where variation exists within the population that needs to be addressed. Defining information that patients want requires attention to detail in order to limit unwarranted assumptions. When wide variation exists in the needs of patients within the population, the PtDA process will require tailoring information presented to the needs of the individual patient.

## List of abbreviations

DA: decision aid; DCS: Decisional Conflict Scale; PtDA: patient decision aid; RCT: randomized controlled trial

## Competing interests

Deb Feldman-Stewart has received travel support to teach a course on designing evidence-based decision aids; Heather L Shepherd has received investigator initiated research funding; and Marla L. Clayman has received research funding from the Informed Medical Decisions Foundation, a not-for-profit (501 (c)3 ) private foundation (http://www.informedmedicaldecisions.org). The Foundation develops content for patient education programs. The Foundation has an arrangement with a for-profit company, Health Dialog, to co-produce these programs. The programs are used as part of the decision support and disease management services Health Dialog provides to consumers through health care organizations and employers.

Mary Ann O’Brien, B. Joyce Davison, Masahito Jimbo, Michel Labrecque, and Richard W. Martin have no competing interests to declare.

## Authors’ contributions

DFS and MAO'B contributed to acquiring and analyzing data. All authors participated in interpretation of the data, revising manuscripts and providing approval of final manuscript.

## Declarations

Publication of this supplement was funded by an unrestricted grant from the Informed Medical Decisions Foundation, by the Department of Oncology at Queen's University (Feldman-Stewart), and the Ontario Institute of Cancer Research (O'Brien and Martin). Administrative and editorial support for this supplement was also provided by The University of Texas MD Anderson Cancer Center.

This article has been published as part of *BMC Medical Informatics and Decision Making* Volume 13 Supplement 2, 2013: The International Patient Decision Aid Standards (IPDAS) Collaboration’s Quality Dimensions: Theoretical Rationales, Current Evidence, and Emerging Issues. The full contents of the supplement are available online at http://www.biomedcentral.com/bmcmedinformdecismak/supplements/13/S2.
